# Comparable Renal Function at 6 Months with Tacrolimus Combined with Fixed-Dose Sirolimus or MMF: Results of a Randomized Multicenter Trial in Renal Transplantation

**DOI:** 10.1155/2010/731426

**Published:** 2010-10-05

**Authors:** Eveline Van Gurp, Jesus Bustamante, Antonio Franco, Lionel Rostaing, Thomas Becker, Eric Rondeau, Zenon Czajkowski, Andrzej Rydzewski, Antonio Alarcon, Petr Bachleda, Jiri Samlik, Dirk Burmeister, Luis Pallardo, Marie-Christine Moal, Boleslaw Rutkowski, Zbigniew Wlodarczyk

**Affiliations:** ^1^Department of Internal Medicine, Erasmus MC, 's-Gravendijksewal 230, 3015 CE Rotterdam, The Netherlands; ^2^Nephrology Service, Hospital Clinico de Valladolid, 47011 Valladolid, Spain; ^3^Nephrology Service, Hospital General de Alicante, 03070 Alicante, Spain; ^4^Nephrology Service, Hospital de Rangueil, Toulouse, France; ^5^Department of General, Vascular, and Transplantation Surgery, Medizinische Hochschule Hannover, Hannover, Germany; ^6^Nephrology and Renal Transplant Service, Hospital Tenon, Paris, France; ^7^Unit of Anesthesiology and Intensive Care, Wojewódzki Szpital Zespolony, Szczecin, Poland; ^8^Department of Internal Diseases and Nephrology, Centralny Szpital Kliniczny MSWiA, Warszawa, Poland; ^9^Nephrology Service, Hospital Son Dureta, Palma de Mallorca, Spain; ^10^Transplant Center, FN a LF UP Olomouc, Olomouc, Czech Republic; ^11^Transplant Center, FNsP Ostrava, Ostrava, Czech Republic; ^12^Urology Clinic, University Hospital Rostock, Rostock, Germany; ^13^Servicio de Nefrologia, Hospital Universitario Dr. Peset, Valencia, Spain; ^14^Nephrology Service, Hospital la Cavale Blance, Brest, France; ^15^Department of Nephrology, Medical University of Gdansk, Gdansk, Poland; ^16^Transplantation Unit, Wojewódzki Szpital Zespolony, Poznan, Poland

## Abstract

In a multicenter trial, renal transplant recipients were randomized to tacrolimus with fixed-dose sirolimus (Tac/SRL, *N* = 318) or tacrolimus with MMF (Tac/MMF, *N* = 316). Targeted tacrolimus trough levels were lower in the Tac/SRL group after day 14. The primary endpoint was renal function at 6 months using creatinine clearance (Cockcroft-Gault) and was comparable at 66.4 mL/min (SE 1.4) with Tac/SRL and at 65.2mL/min (SE 1.3) with Tac/MMF (completers). Biopsy-confirmed acute rejection was 15.1% (Tac/SRL) and 12.3% (Tac/MMF). In both groups, graft survival was 93% and patient survival was 99.0%. Premature withdrawal due to an adverse event was twice as high in the Tac/SRL group, 15.1% versus 6.3%. Hypercholesterolemia incidence was higher with Tac/SRL (*P* < .05) while CMV, leukopenia, and diarrhea incidences were higher with Tac/MMF (*P* < .05). The incidence of any antidiabetic treatment for >30 consecutive days in previously nondiabetic patients was 17.8%, Tac/SRL, and 24.8%, Tac/MMF. Evaluation at 6 months showed comparable renal function using tacrolimus/sirolimus and tacrolimus/MMF regimens.

## 1. Introduction

Tacrolimus combined with various adjunctive agents has been evaluated in clinical trials. Studies have shown that tacrolimus provides good renal allograft function and excellent immunologic protection [1, 2] and combined with MMF has proved to be a highly efficacious regimen in renal transplantation [3, 4] which is now the standard regimen in most transplant centers in Europe and the US. 

Clinical comparisons of efficacy outcomes between tacrolimus with sirolimus and tacrolimus with MMF have demonstrated similarly low incidences of biopsy-confirmed acute rejection (BCAR). The results of one large multicenter European study revealed comparable BCAR rates between regimens using 0.5 mg sirolimus and 1.0 g MMF but significantly lower incidences of BCAR when a 2 mg dose of sirolimus was administered [5]. A randomized clinical trial conducted in the US found low and comparable rates of BCAR using 2.0 mg sirolimus and 2.0 g MMF (13.0% and 11.4%, resp.) [6]. Results of safety analyses in both studies, however, revealed more adverse effects with sirolimus than with MMF, including higher measurements of some indicators of cardiovascular risk.

Combining sirolimus with a calcineurin inhibitor (CNI) may accelerate CNI-induced nephrotoxicity especially in the presence of delayed graft function (DGF) [7]. The results of one clinical trial showed significantly worse renal function with a tacrolimus and sirolimus combination compared with a tacrolimus and MMF combination using sirolimus 2 mg daily [6]. In contrast, a phase II study compared three maintenance doses of sirolimus (0.5 mg, 1 mg, and 2 mg daily) combined with tacrolimus against a standard tacrolimus and steroid regimen and found no difference in serum creatinine [8].

In this multicenter, randomized, clinical trial, we used renal function, as measured by calculated creatinine clearance at 6 months, to compare tacrolimus combined with sirolimus against a tacrolimus and MMF control. Our secondary objective was to compare the efficacy and safety profiles of the two regimens. Unlike other studies, sirolimus was administered in a fixed dose, as opposed to a concentration-controlled dosing concept, and a maintenance dose of 1.0 mg sirolimus was initiated after 28 days.

## 2. Patients and Methods

This was a 6-month, randomized, phase III clinical trial conducted in 51 centers in 13 European countries. The study was performed in accordance with the ethical principles described in the amended Declaration of Helsinki following approval from the institutional review committee at each participating center. Patients provided written informed consent prior to study randomization. The study was conducted between October 2004 and July 2006.

Patients aged 18 to 60 undergoing primary renal transplantation or retransplantation (unless the graft was lost due to rejection within the previous 12 months) from a deceased or living donor were eligible for study enrollment. Excluded from the study were patients with high immunological risk (defined as having a panel reactive antibody grade >50% in the previous 6 months and/or having a previous graft survival <1 year due to immunological reasons). Other exclusion criteria included continuously elevated tests of liver function, patient or donor was HIV positive, previous recipient of an organ transplant other than kidney, organ cold ischemia time >30 hours, intolerance to any of the study drugs or the requirement of additional immunosuppressive drugs or antibodies, malignancy, severe hypercholesterolemia (>350 mg/dL or 9.1 mmol/dL), and uncontrolled infection. Patients provided written informed consent before study enrollment. 

Patient randomization was 1:1, stratified by center and occurred before the first dose of study medication was administered. Sealed randomization envelopes were supplied by the study sponsor. Patients were assigned to treatment with tacrolimus, sirolimus, and steroids (Tac/SRL) or tacrolimus, mycophenolate mofetil, and steroids (Tac/MMF). 

Tacrolimus was administered in both treatment groups at an initial daily dose of 0.2 mg/kg administered twice (one dose preoperatively and one dose postoperatively). Recommended trough levels for tacrolimus in both groups on days 0 to 14 were 10–15 ng/mL. As the combination of a CNI and sirolimus has been shown to be potentially more nephrotoxic than a CNI and MMF [5, 8–10], recommended tacrolimus trough levels were lower after day 14 in the Tac/SRL group to minimize toxicity. Trough levels were set at 4–8 ng/mL on days from 15 to 42 and 4–6 ng/mL on days from 43 to 183. Recommended trough levels in the Tac/MMF group were set higher at 8–12 ng/mL on days from 15 to 42 and 5–10 ng/mL on days from 43 to 183. 

A loading dose of sirolimus 6.0 mg was administered with the postoperative dose of tacrolimus and was followed by maintenance doses of 2.0 mg for 28 days and 1.0 mg thereafter. A loading dose of MMF 1.0 g was administered pretransplant followed by a daily dose of 2.0 g for the first 14 days and 1.0 g daily thereafter. Doses of sirolimus and MMF could be reduced or suspended due to medication side effects for no more than 21 days. 

Adjuvant treatment with corticosteroids was permitted in both regimens using a 100–500 mg bolus dose given peri-operatively and a 125 mg bolus on day 1. Thereafter, steroids were to be steadily tapered from 20 mg on day 2 to 5 mg by day 90 and discontinued on day 91.

First line treatment for acute rejection was corticosteroids administered according to local practice; antibody administration was permitted if a biopsy revealed a severe vascular rejection (Banff IIb or III). If rejection was refractory to corticosteroids, then treatment with OKT3 or polyclonal antibodies according to local practice was permitted. Other systemic immunosuppressive medications were prohibited.

Prophylactic treatment for *Pneumocystis carinii* pneumonia consisting of cotrimoxazole was required throughout the study. Prophylactic antiviral treatment for CMV in cases where a CMV-positive donor graft was transplanted in a CMV-negative recipient consisting of gancyclovir or equivalent was required.

### 2.1. Outcome Assessments

The primary efficacy endpoint was renal function as measured by calculated creatinine clearance at month 6 (Cockcroft-Gault formula [11]). Since the planning of this study, the Modification of Diet in Renal Disease formula (MDRD) has become increasingly used to estimate glomerular filtration rate following renal transplantation; hence, a post hoc analysis of the primary endpoint was run using the MDRD-4 [12]. Secondary endpoints included the incidence and time to clinical acute rejection and BCAR, patient and graft survival, incidence of adverse events, absolute change in serum lipids, renal dysfunction, and the incidence of diabetes mellitus and hypertension. 

The outcomes measured in the study were defined as follows. Graft loss: retransplantation, nephrectomy, death or dialysis ongoing at study end or patient withdrawal. DGF: postoperative dialysis for more than one day during the time period from day 0 to day 7. Hypertension: systolic blood pressure >140 mmHg or diastolic blood pressure >90 mmHg within one measurement. Diabetes mellitus: antidiabetic treatment for >30 consecutive days. Safety assessments included the monitoring of adverse events and vital signs as well as clinical laboratory evaluations. Adverse events were coded using MedDRA (version 8.0). 

A total of seven scheduled assessment visits took place. Patients were regularly monitored for adverse events.

### 2.2. Statistical Analysis

A difference in the mean creatinine clearance of 7.5 mL/min was considered a clinically meaningful margin of noninferiority. Therefore, it was calculated that 225 patients would be required per treatment group to conclude non-inferiority with a power of at least 90%. With an assumed dropout rate of 25%, 600 patients were to be randomized, 300 patients per treatment group. A test for superiority was to be performed without further adjustment of the level of significance if non-inferiority was shown.

The full analysis set (FAS), all patients who were randomized, transplanted, and received at least one dose of study medication, was used for analysis. Data from patients belonging to the FAS who completed the study were used to analyze the primary endpoint. Because of the potential bias using observed cases only, a sensitivity analysis of the primary endpoint was performed on all patients with data available at 6 months. Missing values of creatinine clearance were not imputed. 

The incidence and time to acute rejection as well as patient and graft survival were analyzed using Kaplan-Meier methods. The difference between treatment groups was analyzed using the Wilcoxon-Gehan test. Two-sided 95% confidence limits for the difference in survival at month 6 were calculated using normal approximation with the variance calculated according to the Greenwood formula. Secondary endpoint variables were analyzed using descriptive statistics. The incidence of adverse events was compared between treatment groups using descriptive *P*-values of Fisher's exact test.

## 3. Results

### 3.1. Patients

In total, 659 patients were enrolled in the study. Of these, 634 underwent transplantation and received at least one dose of study medication (tacrolimus, sirolimus, or MMF), and thus were eligible for the FAS. The treatment groups were comparable with respect to demographic and baseline characteristics. The population was European, predominantly Caucasian with a mean age in the mid-40s, low immunologic risk, with few comorbidities (Table 1). 

**Figure 1:**
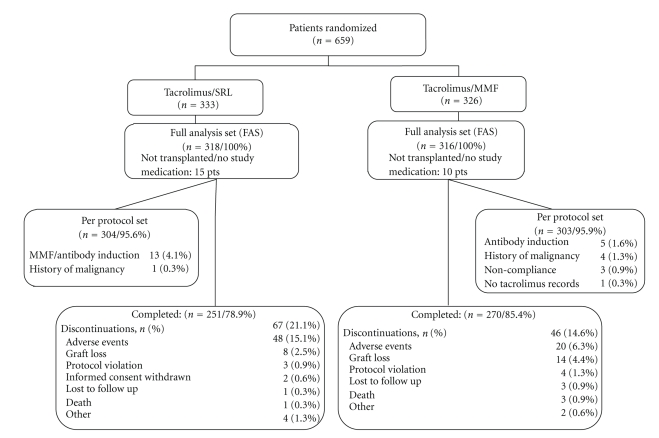
The full analysis set, which consisted of all patients who received at least one dose of study drug, included 318 patients in the Tac/SRL group and 316 patients in the Tac/MMF group. More patients in the Tac/SRL group withdrew from the study prematurely, the majority because of an adverse event.

Approximately 79% of all randomized patients completed the study (Figure 1). The most common reason for study withdrawal in both groups was an adverse event. 

### 3.2. Immunosuppression

**Figure 2:**
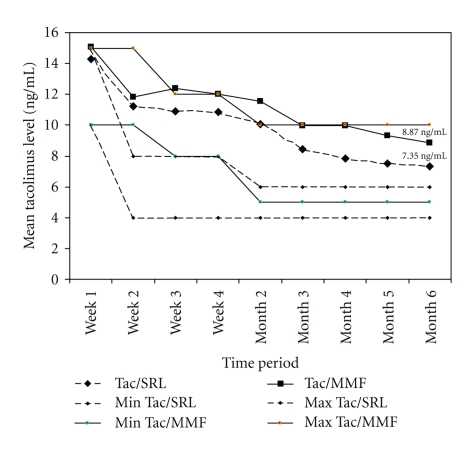
Data presented are for the FAS population. Mean tacrolimus trough levels in the Tac/SRL group were higher than the recommended upper level throughout the study. In the Tac/MMF group, mean trough levels were within the recommended ranges from month 3 onward.

Protocol-defined targeted tacrolimus trough levels were different for the two groups after day 14. Despite lower targeted levels after day 14 in the Tac/SRL group, mean trough levels remained above the upper recommended range throughout the study. At month 6, targeted trough level was 4–6 ng/mL, whereas the observed mean level was 7.4 ng/mL (Figure 2). In the Tac/MMF group, mean tacrolimus trough level at month 6 was 8.9 ng/mL which was within the targeted range of 5–10 ng/mL. Mean (±SD) daily dose of tacrolimus was similar between the groups at 6 months: 4.9 mg (±3.1) in the Tac/SRL group and 5.2 mg (±2.9) in the Tac/MMF group. 

The mean (SD) daily administered dose of sirolimus steadily decreased from 1.7 mg (±0.5) at day 28, to 1.0 mg (±0.2) by day 61, and 1.0 mg (±0.2) by month 6 (median dose 1.0; range, 0.3–2.0 mg). Similarly, the mean daily dose of MMF decreased from 1.9 g (±0.2) during week 2 to 1.3 g (±0.4) at week 3 and 1.0 g (±0.3) at 6 months (median dose 1.0; range 0.3–2.0 g). There were no patients in either treatment group who violated protocol and discontinued the adjunct immunosuppressant for >21 days or switched adjunct immunosuppressant. Four patients in the Tac/MMF group completely discontinued taking MMF. 

Adherence to the protocol-defined withdrawal of steroids at month 3 was low in both groups. At month 4, approximately half of the patients in each group were still taking steroids. By month 5 the numbers of patients maintained on steroids dropped dramatically in both groups. This was 81/318 patients (25.5%) in the TAC/SRL group taking maintenance steroids at a median daily dose of 5.0 mg (range, 1.3–82.5) and 99/316 patients (31.3%) in the TAC/MMF group taking maintenance steroids at a median daily dose of 5.0 mg (range, 1.4–36.3). At month 6, maintenance steroids were being taken by 59/318 patients (18.6%) in the Tac/SRL group at a median daily maintenance dose of 5.0 mg (range, 1.3–25.0) compared with 84/316 patients (26.6%) in the Tac/MMF group taking a median daily maintenance dose of 5.0 mg (range, 0.6–30.0).

### 3.3. Renal Function

The calculated mean creatinine clearance values at 6 months were similar in the two groups. In 250/318 patients (completers) in the Tac/SRL group, the mean creatinine clearance was 66.4 mL/min (SE 1.4), and in 266/316 patients (completers) in the Tac/MMF group, it was 65.2 mL/min (SE 1.3) (Table 2). The lower boundary of the two-sided 95% CI for the difference in mean creatinine clearance was −2.58 mL/min which is well within the predefined margin of −7.5 mL/min. Hence, Tac/SRL was noninferior to Tac/MMF. The test of superiority of Tac/SRL over Tac/MMF was not significant. Results of the post hoc analysis using the MDRD-4 formula to estimate renal function showed no meaningful difference between the two treatments although it was notable that mean estimated GFR as measured by MDRD was lower than calculated creatinine clearance using the Cockcroft-Gault formula in both groups.

Delayed graft function was reported in 64/318 patients (20.1%) in the Tac/SRL group and in 52/316 patients (16.5%) in the Tac/MMF group. Of these patients, dialysis was required by 53 patients (16.7%) in the Tac/SRL group for a median of 10 days (range: 2–76 days) and by slightly fewer patients 45 (14.2%) in the Tac/MMF group for a median of 8 days (range 2–26). There was no difference between the treatment groups in renal function as measured by mean calculated creatinine clearance in patients with and without DGF (Table 2). Mean serum creatinine was numerically better in patients treated with Tac/SRL with notably better results in the subgroup of patients without DGF.

### 3.4. Acute Rejection

The overall frequency of acute rejection diagnosed by either clinical signs, symptoms, or biopsy-confirmed was similar in the treatment groups (Table 3). The protocol specified that biopsies were to be performed if the patient displayed signs and symptoms of rejection; however, biopsies were not performed on all suspected rejections. Incidence of BCAR was 15.1% in the Tac/SRL group and 12.3% in the Tac/MMF group. There were no differences found in either the histological classification of biopsy specimens or in the clinical course of the rejection episodes.

**Figure 3:**
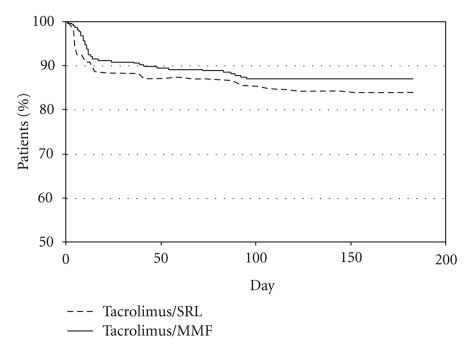
Estimated rate of patients free from biopsy-confirmed acute rejection (Kaplan-Meier method). The estimated rate of patients free from BCAR (Kaplan-Meier method) at 6 months was 83.8% in the Tac/SRL group and 87.1% in the Tac/MMF group (difference between groups −3.0%; 95% confidence interval: −8.9% to 2.4% [Greenwood formula]).

The overall estimated rate of patients free from BCAR at 6 months (Kaplan-Meier method) was 83.8% in the Tac/SRL group and 87.1% in the Tac/MMF group (difference between groups in 6-month survival was −3.0%; 95% confidence interval: −8.9% to 2.4% [Greenwood formula]). More than half of the BCARs in each group occurred during the first two weeks after transplant (Figure 3). In the Tac/SRL group nearly half of BCAR episodes (23/48) occurred during week one whereas in the Tac/MMF group nearly half of BCAR episodes (19/39) occurred during week two. During month 3, the number of patients experiencing a BCAR was similar, (2/48 in the Tac/SRL group and 4/39 in the Tac/MMF group) whereas acute rejection during month 6 was higher in the Tac/SRL than in the Tac/MMF group (7/48 versus 2/39 patients, resp.).

### 3.5. Patient and Graft Survival

Patient and graft survival were equivalent between treatment groups (Table 3). Kaplan-Meier estimate for overall patient survival rate was 99.0% (95% CI: 98.0% to 100%) at month 6 for both treatment groups. During the study, 4 patients died: 1 patient in the Tac/SRL group and 3 patients in the Tac/MMF group. The cause of death of the patient in the Tac/SRL group was hyperkalemia and the cause of death for all 3 patients in the Tac/MMF group was cardiorespiratory; no deaths were assessed by the investigator to have been related to the study medication. Two patients died in the Tac/SRL group after their withdrawal from the study: the cause of death in both cases was a surgical complication.

Kaplan-Meier estimates of graft survival rates at month 6 were 92.7% (Tac/SRL [95% CI: 89.8% to 95.6%]) and 93.3% (Tac/MMF [95% CI: 90.5% to 96.1%]). Graft loss due to long-term dialysis or transplantectomy occurring during the study was comparable at 17/318 patients (5.3%) in the Tac/SRL group and 16/316 patients (5.1%) in the Tac/MMF group.

### 3.6. Safety

There was no difference in the overall incidence of adverse events which was approximately 93% in each treatment group. A significant difference in the occurrence of several adverse events was found (Table 4). Hypercholesterolemia and peripheral edema occurred significantly more often in patients in the Tac/SRL group (*P* < .05, Fisher's exact test). Conversely, hyperkalemia, CMV infection, nasopharyngitis, leukopenia, and diarrhea occurred significantly more often in patients in the Tac/MMF group (*P* < .05 and *P* < .001 for CMV infection and leukopenia, Fisher's exact test for all comparisons). 

Twice as many patients receiving Tac/SRL withdrew due to an adverse event (48/318 [15.1%]) than patients who received Tac/MMF (20/316 [6.3%]). By body system (MedDRA coded), the most commonly reported adverse event leading to study withdrawal in both groups was a renal or urinary disorder (by preferred term these were renal artery/renal vein thrombosis, renal artery stenosis, renal infarct, and renal tubular necrosis). 

Similar percentages of patients in the Tac/SRL and Tac/MMF groups experienced serious adverse events, 45.9% (146/318) and 43.4% (137/316), and similar percentages of patients experienced adverse events that were assessed by the investigator to be causally related to the study medication, 69.2% (220/318) and 67.7% (214/316), respectively. Two patients in each group were diagnosed with a malignancy during the study. In the Tac/SRL group, 1 patient was diagnosed with lymphoproliferative disease and 1 with an abdominal tumor. In the Tac/MMF group 2 patients were diagnosed with a renal carcinoma. 

Changes in mean serum lipid values were negligible between baseline and month 6 in both treatment groups (Table 5). There were no differences found between the groups in the incidence of hypertension and approximately three-quarters of patients in both groups were receiving treatment for hypertension at study completion. The incidence of *de novo* diabetes mellitus was lower in the Tac/SRL than in the Tac/MMF group. 

No significant differences between groups were observed in any mean hematology value (Table 5). As classified by the investigator, the incidence of viral infections was significantly higher in the Tac/MMF group: 10.4% compared with 19.6% (*P* = .001, Fisher's exact test).

The duration of initial hospitalization as well as the number of episodes of subsequent hospitalizations was similar between the two groups.

## 4. Discussion

This large multicenter study investigated the non-inferiority of tacrolimus combined with fixed-dose sirolimus against a standard regimen of tacrolimus combined with MMF by measuring creatinine clearance at 6 months in renal transplant patients. Results showed that a tacrolimus and sirolimus regimen is noninferior to the widely used combination of tacrolimus and MMF. 

There was no apparent difference in renal function when comparing groups receiving the two different tacrolimus-based combinations. The phase II comparative trial which provided the basis for our trial showed no indication of an influence of sirolimus on renal function when combined with tacrolimus using fixed doses of sirolimus (0.5 mg 1.0 mg or 2 mg) for 6 months [8], and serum creatinine levels in the sirolimus group in our study were 14% lower than those reported using tacrolimus combined with sirolimus 1 mg [5]. The similar results in renal function we observed contrast with results from a phase III clinical trial in which both serum creatinine and creatinine clearance were better with MMF than with sirolimus at 6 months [6], a trend which continued and was reported in the one-year results of that study [13]. Analysis of retrospectively collected Scientific Renal Transplant Registry (SRTR) data showed worse graft survival at 1 year with a tacrolimus/sirolimus combination (91.8%) versus a tacrolimus/MMF combination (94.2%) with differences in graft survival at 3 years reaching statistical significance (80.3%, tacrolimus/sirolimus versus 85.9%, tacrolimus/MMF) [7]. Decreasing the doses of the adjuvant agents early in the posttransplant phase and lowering tacrolimus exposure may have influenced our results. We did find numerically higher rates and a longer duration of DGF with Tac/SRL than with Tac/MMF. Admittedly, caution should be applied before drawing any conclusion on the benefit on renal function of one regimen over the other as used in this study. We acknowledge that the 6-month study duration makes it difficult to project renal function or graft survival longer-term and the study design makes it difficult to identify variables which might abate or accelerate nephrotoxicity. 

For the primary analysis, we used data from study completers. While an analysis using imputation to account for missing data might yield additional information, it is unlikely that this procedure would provide a different study conclusion. As shown in Table 2, the difference in creatinine clearance between “completers” and “patients with month 6 data” is minimal. We suspect that the inclusion of imputed data would not change the observed difference in creatinine clearance between the treatment groups; the confidence interval, however, would be affected becoming wider due to increased standard deviations or narrower due to increased sample size with the inclusion of imputed missing values.

Using the experience gained from a phase II trial [8], we implemented a novel approach to dosing sirolimus using two stages to reduce the fixed-dose (i.e., 2 mg sirolimus for the first 28 days and 1 mg/day thereafter). Protocol-defined trough levels for tacrolimus were lower in the Tac/SRL group, an approach supported by clinical research findings [5, 14]. Dosing of MMF and tacrolimus in the Tac/MMF group was based on the favorable results of previous studies [15, 16]. The intent of both treatment schemas was to achieve a balance between protection from acute allograft rejection and acceptable tolerability. 

Several aspects of regimen tolerability observed in this study are worth mentioning. Firstly, no patient in either treatment group switched the adjuvant immunosuppressant and only four patients in the MMF group discontinued this drug. These results contrast results of a previous study in which a significantly higher rate of drug discontinuation was observed with tacrolimus and sirolimus compared to tacrolimus and MMF (21.1% versus 10.8%) [6]. Secondly, adherence to protocol-defined steroid withdrawal was disappointing with only 50% of patients in each group steroid-free by month 4. This finding together with the short follow up after steroid discontinuation makes it inappropriate to comment on the safety of withdrawing steroids at 3 months. 

The third aspect of regimen tolerability which should be mentioned is the higher tacrolimus exposure throughout the study in the Tac/SRL group. Although tacrolimus levels in this study were 19% lower than what was reported in the phase II study [8], the levels may nonetheless reflect hesitancy among clinicians to reduce tacrolimus dose in the absence of steroids despite potential nephrotoxicity when administering a CNI and mTOR combination. Tacrolimus levels were also reported to be at the upper end of the target range with low dose tacrolimus in the large, randomized multicenter ELITE-Symphony study [17]. 

The incidences of BCAR we report are in line with prospective study data with incidences ranging from 12% to 24% [15, 17–19] and retrospective review data reporting an incidence of 17% using immunosuppressive regimens without antibody induction [20]. Further, rates of BCAR in the Tac/SRL group are comparable to those reported in studies in which sirolimus doses remained constant for 6 months (15.7%) [5]. Although incidences of clinical rejection in our study were similar, not all episodes of clinical rejection were verified by biopsy as was specified in the protocol. Our results indicate that it may be feasible to use a fixed sirolimus dose reduction schema without compromising efficacy; this, however, remains to be proven in prospective trials. 

The incidence and type of adverse events reported during the study reflect the known safety profile of both adjunctive therapies (sirolimus and MMF). Study results do not provide clear conclusions in terms of a benefit on safety outcomes of one regimen over the other. Known side effects of MMF therapy, leucopenia, and subsequent infections including CMV infection [13, 21] were more frequently reported with Tac/MMF. Lastly, we observed greater premature study withdrawal in the Tac/SRL than in the Tac/MMF group, as was also reported in another European randomized study [5]. The withdrawal rates in that study (10.5% with sirolimus versus 4.9% with MMF) were somewhat lower than the rates we report. 

In conclusion, results of this study show similarly good renal function at 6 months in renal transplant recipients when tacrolimus is combined with either sirolimus or with MMF. The use of a novel fixed-dose dosing schema for sirolimus was not associated with increased risk of rejection. Both tacrolimus-based regimens provided comparable benefit in this study population.

##  Authorship

All named authors commented on the study design, took part in the study, and reviewed and approved the final manuscript.

##  Funding Sources

The study was funded by Astellas Pharma Europe Ltd, London, UK. The study sponsor was involved in the design of the study, analysis of the data, and preparation of the manuscript. 

##  Conflict of Interests

The authors' departments received study grants from Astellas Pharma GmbH (now Astellas Pharma Europe Ltd.) to perform the study. E. Van Gurp, L. Rostaing, T. Becker, E. Rondeau, Z. Czajkowski, A. Alarcon, L. M. Pallardo, B. Rutkowski, and Z. Wlodarczyk. have received fees/sponsorship for speaking/chairing meetings on behalf of Astellas. All other authors declare that they have no conflict of interest.

## Figures and Tables

**Table 1:** Patient Demographics and Baseline Characteristics.

	Tacrolimus/Sirolimus *N* = 318	Tacrolimus/MMF *N* = 316
*Recipients*		
Age, mean (SD), y	44.3 (11.3)	44.9 (11.1)
Range, y	18–66	18–72
Male, Number (%)	204 (64.2)	204 (64.6)

Race, Number (%)		
Caucasian	299 (94.0)	303 (95.9)
Black	10 (3.1)	7 (2.2)
Oriental	7 (2.2)	4 (1.3)
Other	2 (0.6)	2 (0.6)

Previous transplants, Number (%)		
0	306 (96.2)	301 (95.3)
1	12 (3.8)	14 (4.4)
3	0 (0.0)	1 (0.3)
PRA^a^: 0%–50%, Number (%)	316 (99.7)	313 (100.0)
50%–100%, Number (%)	1 (0.3)	0 (0.0)

*Donors*		
Donation type: Number (%)		
Living	41 (12.9)	32 (10.1)
Deceased	277 (87.1)	284 (89.9)

*Transplant*		
ABO identical, Number (%)	296 (93.1)	295 (93.4)
Cold ischemia time, mean (SD), h	16.1 (6.1)	16.0 (5.8)
Mean total HLA mismatch	2.9	3.0
CMV status: Donor +/Recipient −, Number (%)	47 (14.8)	57 (18.0)

FAS

^a^PRA not recorded for all patients.

**Table 2:** Measurements of renal function at month 6.

	*n*	Tacrolimus/Sirolimus *N* = 318	*n*	Tacrolimus/MMF *N* = 316
Creatinine Clearance (Cockcroft-Gault), mean (SE), mL/min				
Patients completing the study	250	66.4 (1.4)	266	65.2 (1.3)
Patients with month 6 data^a^	289	64.6 (1.3)	289	63.7 (1.3)
Patients with DGF	48	56.1 (20.0)	38	60.5 (22.9)
Patients without DGF	241	66.3 (21.5)	251	64.2 (21.9)

Creatinine Clearance (MDRD-4), mean (SD), mL/min				
Patients completing the study	250	55.3 (1.2)	266	54.0 (1.2)
Patients with month 6 data^a^	289	53.9 (1.1)	289	53.0 (1.2)

Serum Creatinine, mean, (SD), *μ*mol/L				
Patients completing the study	250	133.9 (51.4)	266	137.2 (52.7)
Patients with month 6 data^a^	289	138.2 (57.7)	289	144.7 (93.7)
Patients with DGF	48	169.9 (77.0)	38	151.5 (54.8)
Patients without DGF	241	131.9 (51.0)	251	143.7 (98.3)

FAS

DGF: Delayed graft function. MDRD: Modification of Diet in Renal Disease.

^a^Completers and withdrawn patients with available data at 6 months after transplantation.

**Table 3:** Selected secondary endpoints.

	Tacrolimus/Sirolimus *N* = 318	Tacrolimus/MMF *N* = 316
Acute Rejection, Number (%)	82 (25.8)	77 (24.4)

Biopsy confirmed acute rejection, Number (%):	48 (15.1)^a^	39 (12.3)^a^
Spontaneously resolving	1 (0.3)	0 (0.0)
Corticosteroid sensitive	34 (10.7)	32 (10.1)
Corticosteroid resistant	14 (4.4)	8 (2.5)
Resolved with further treatment	12 (3.8)	8 (2.5)
Other	1 (0.3)	0 (0.0)

Histological grade, Number (%):		
Mild (Banff I)	30 (9.4)	20 (6.3)
Moderate (Banff II)	17 (5.3)	17 (5.4)
Severe (Banff III)	1 (0.3)	2 (0.6)

Patient survival^b^, %	99.0	99.0

Graft survival^b^, %	92.7	93.3

FAS

^a^More than one BCAR episode was reported for some patients.

^b^Kaplan-Meier estimates.

**Table 4:** Commonly reported^a^ adverse events—number (%) of patients.

	Tacrolimus/Sirolimus *N* = 318	Tacrolimus/MMF *N* = 316
*Adverse event*		
Metabolism or nutrition	167 (52.5)	156 (49.4)
Hyperglycemia	38 (11.9)	46 (14.6)
Diabetes mellitus	25 (7.9)	32 (10.1)
Hyperkalemia^b^	15 (4.7)	28 (8.9)
Hypercholesterolemia^b^	35 (11.0)	18 (5.7)
Infections	149 (46.9)	162 (51.3)
Urinary tract	83 (26.1)	83 (26.3)
Cytomegalovirus^c^	9 (2.8)	38 (12.0)
Nasopharyngitis^b^	7 (2.2)	19 (6.0)
Blood and lymphatic system^b^	76 (23.9)	102 (32.6)
Anemia	52 (16.4)	68 (21.5)
Leukopenia^c^	5 (1.6)	27 (8.5)
Gastrointestinal disorders	83 (26.1)	90 (28.5)
Diarrhea^b^	38 (11.9)	57 (18.0)
Vascular disorders	74 (23.3)	54 (17.1)
General or site of drug administration	65 (20.4)	50 (15.8)
Peripheral edema^b^	22 (6.9)	10 (3.2)
Nervous system	44 (13.8)	45 (14.2)
Musculoskeletal	42 (13.2)	31 (9.8)

*Serious Adverse Event*		
Infection	48 (15.1)	37 (11.7)
Cytomegalovirus infection^c^	3 (0.9)	16 (5.1)
Vascular disorders	23 (7.2)	14 (4.4)
Gastrointestinal disorders	17 (5.3)	16 (5.1)
Cardiac disorders^b^	2 (0.6)	10 (3.2)

FAS

^a^Listed are the most common adverse events occurring in ≥10% of patients or with a significant difference between groups. Serious adverse events are listed if they occurred in ≥5% of patients. Reports of renal dysfunction/impairment, surgical complications, and abnormal laboratory values are not presented here as an adverse event.

^b^ *P* < .05. ^c^ *P* < .001 (Fisher's exact test for all comparisons).

**Table 5:** Clinical laboratory results, concomitant medications, and safety results.

	*n*	Tacrolimus/Sirolimus *N* = 318	*n*	Tacrolimus/MMF *N* = 316
Total cholesterol, mean (SD), mmol/L	285	5.2 (1.4)	291	5.0 (1.1)
*Change from baseline *	241	*0.5 (1.5)*	240	*0.3 (1. 5) *
LDL, mean (SD), mmol/L	248	3.0 (1.0)	255	2.9 (1.0)
*Change from baseline *	178	*0.3 (1.1)*	183	*0.2 (1.1)*
HDL, mean (SD), mmol/L	258	1.4 (0.5)	265	1.3 (0.4)
*Change from baseline *	192	*0.1 (0.5)*	195	*0.0 (0.4)*
Triglycerides, mean (SD), mmol/L	285	2.3 (1.6)	291	2.1 (1.5)
*Change from baseline *	245	*0.2 (1.5)*	244	−*0.1 (1.7)*

Hematology, mean (SD):				
WBC, ×10^9^/L	314	7.3 (3.1)	313	6.7 (2. 7)
Platelets, ×10^9^/L	314	238.7 (81.8)	313	231.9 (74.9)

*De novo* diabetes mellitus^a^, Number (%)	287	51 (17.8)	278	69 (24.8)
*De novo* diabetes mellitus^b^, Number (%)	287	5 (1.7)	278	20 (7.2)

FAS

^a^Patients without pre-existing diabetes mellitus treated with antidiabetic medications for >30 consecutive days at any time during the study. ^b^Patients without pre-existing diabetes mellitus treated with antidiabetic medications for >30 consecutive days and still receiving this treatment at end of study.
